# Supplementary material for: Evaluating strategies for reversing CRISPR-Cas9 gene drives
Source: Sci Rep. 2017 Sep 8;7:11038. doi: 10.1038/s41598-017-10633-2 (PMC5591286; doi:10.1038/s41598-017-10633-2)
Supplement: Supplementary file 1 — Supplemental info [file 41598_2017_10633_MOESM1_ESM.pdf]

Evaluating strategies for reversing CRISPR-Cas9 gene drives: Supplemental Information

Michael R. Vella[1,2], **Christian E. Gunning**[3], **Alun L. Lloyd**[1,2], **and Fred Gould**[2,3,*]

[1]North Carolina State University, Biomathematics Graduate Program, Department of Mathematics, Raleigh, 27695, USA
[2]North Carolina State University, Genetic Engineering and Society Center, Raleigh, 27695, USA
[3]North Carolina State University, Department of Entomology and Plant Pathology, Raleigh, 27695, USA
[*]fred_gould@ncsu.edu

Note 1

We examine the qualitative behavior of the system for various fitness costs and assumptions. Figure S1 shows different qualitative behavior present with specific sets of parameter values by using phase plots, which illustrate the change in allele frequencies in one generation for a grid of initial conditions. The allele frequencies are displayed in DeFinetti diagrams[1], where the sum of the frequencies at each point adds to 1. The qualitative difference between the RD and IRD seen in Figures 1 and 2 is visible in Figure S1a (RD) and S1c (IRD), which have parameter values identical to those in Figure 2. Increasing the fitness cost to individuals with a copy of both the HD and countermeasure ($s_{HD/C}$) changes the qualitative behavior of the system. For the RD, with $s_{HD/C} = 0.6$, trajectories spiral outward, toward the edges of the system (Fig. S1b). For the IRD, increasing $s_{HD/C}$ to 0.9 demonstrates bistability, where the system can reach fixation of either the IRD or the HD, depending on initial conditions (Fig. S1d).

To consider a range of values of s_{HD}, s_C, and $s_{HD/C}$, equilibria and their stabilities were computed numerically. Stability was calculated by finding the eigenvalues (λ_i) of the Jacobian of the system evaluated at that equilibrium, and checking if $|\lambda_i| \leq 1$ for all i. We assumed perfect homing ($e_{HD} = e_C = 1$) such that $q_R = 0$ at all times, and recessive fitness costs in wild-type heterozygotes ($h_{HD} = h_C = 0$) in Figures S2-S3 and additive fitness costs in wild-type heterozygotes ($h_{HD} = h_C = 0.5$) in Figures S4-S5.

Different equilibria are stable for different regions of parameter values, as indicated by color in Figures S2 and S4. For each of the three countermeasures, Figures S2 and S4 illustrate the long-term behavior seen for different values of the HD fitness cost (s_{HD}), HD/C heterozygote fitness cost ($s_{HD/C}$) and the countermeasure fitness cost (s_C). Each row of the figure depicts a different countermeasure (SR, RD or IRD), and different panels across a row depict different countermeasure fitness costs. Within each panel, the HD fitness cost is shown on the horizontal axis and the HD/C heterozygote fitness cost on the vertical. Using HD, C, and W as abbrevations for homing drive, countermeasure, and wild-type alleles, respectively, the colors indicate the stable equilibrium as follows: red for HD fixation, blue for C fixation, green for an equilibrium that consists of a combination of C and W (i.e., no HD at equilibrium), purple for an equilibrium with a combination of HD and C, orange for an equilibrium with a combination of HD, C, and W, and yellow for bistability, when two of the previously listed equilibria are both stable. For SR and RD countermeasures, the bistable regions have both HD fixation and a combination of C and W as stable equilibria, with initial conditions determining the long-term behavior of the system. For the IRD, the bistable regions have both HD and IRD fixation as stable equilibria, as shown in the example of Figure S1d. The difference for the IRD is because unlike the SR and RD, the IRD maintains a relative fitness advantage over W even in the absence of the HD. Finally, the brown regions indicate that those parameter values do not result in any stable equilibria with valid frequencies (i.e., each frequency in range [0,1]). Such cases often result in oscillatory dynamics away from an unstable, polymorphic equilibrium, as depicted in Figure S1b.

Figures S3 and S5 correspond to Figures S2 and S4, respectively, and show the HD frequency at equilibrium. The empty areas of the figures indicate that the system does not always reach the same equilibrium (i.e., bistable or no stable). The SR and RD countermeasures only have stable equilibria without HD present when the countermeasure fitness cost is 0 ($s_C = 0$). The IRD, on the other hand, often has a stable equilibrium without any HD. In regions where there are intermediate frequencies of HD at equilibrium, the equilibrium HD frequency tends to increase as countermeasure fitness decreases. This explains the decrease in minimum HD frequency with decreasing countermeasure fitness costs shown in Figures 3 and 4, since oscillations around smaller frequencies of HD must reach small HD frequencies.

References

[1]Hoppensteadt, F. C., & Peskin, C. (2013). Mathematics in medicine and the life sciences (Vol. 10). Springer Science & Business Media.

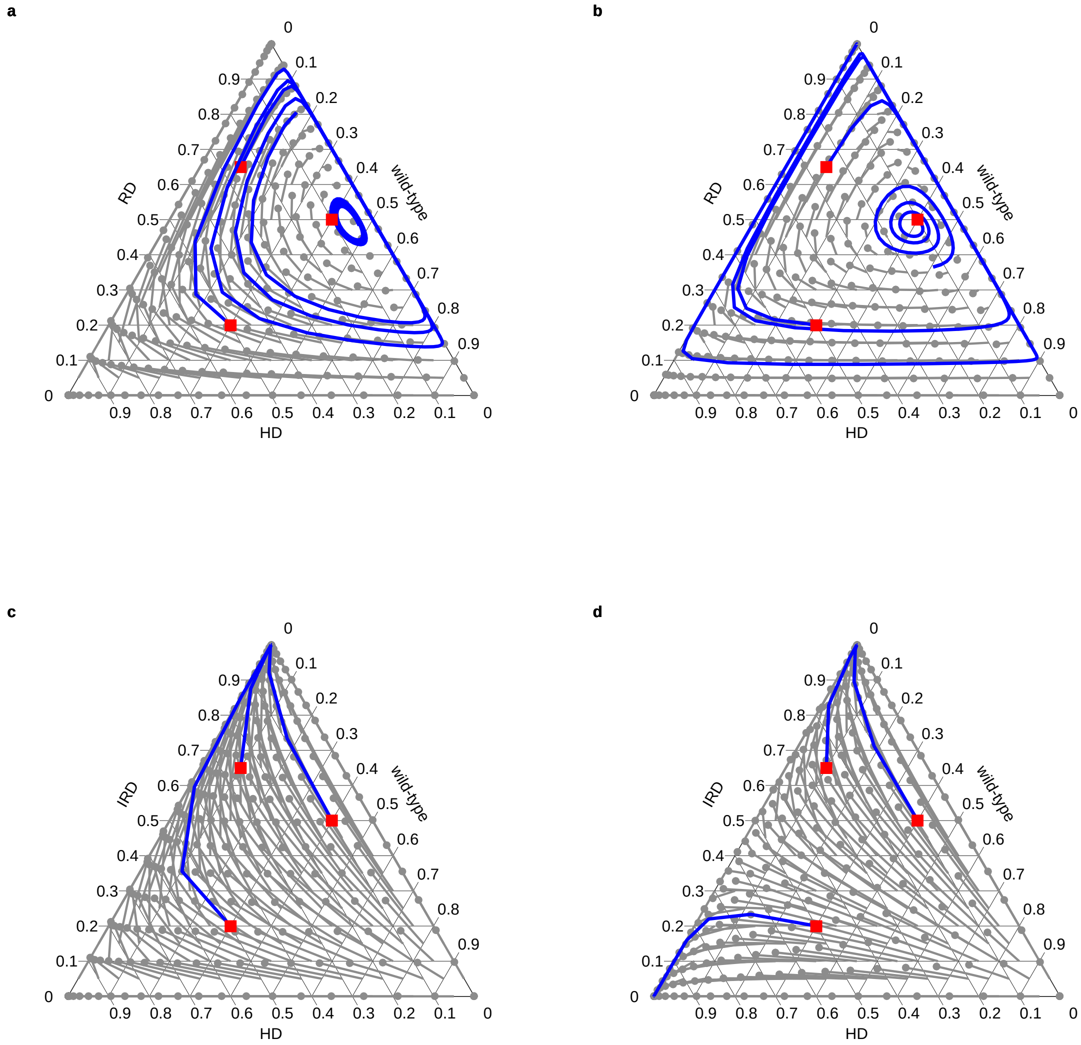

Figure S1. Phase plots, showing change in allele frequencies in one generation for a grid of initial conditions, for RD (a,b) and IRD (c,d). DeFinetti diagrams[1] show allele frequencies with black lines showing the allele frequency change in one generation, going toward the dot. Blue lines follow separate trajectories for 100 generations, starting from the red squares. **a/c)** parameter values from Figure 2 ($s_{HD} = 0.3$, $s_C = s_{HD/C} = 0.2$) demonstrate the stable polymorphic equilibrium for the RD and countermeasure fixation for the IRD. **b)** RD system with increased heterozygote fitness cost ($s_{HD/C} = 0.6$), resulting in no stable equilibria with frequencies in range [0,1] (brown region in Fig. S2). Trajectories oscillate away from the unstable, polymorphic equilibrium. **d)** IRD system with increased heterozygote fitness cost ($s_{HD/C} = 0.9$), resulting in a bistable system (yellow region in Fig. S2). Some initial conditions lead to HD fixation and others lead to countermeasure fixation. Note that near the edge of the triangle, stochastic loss of the allele with small frequency becomes likely in a finite, randomly mating population.

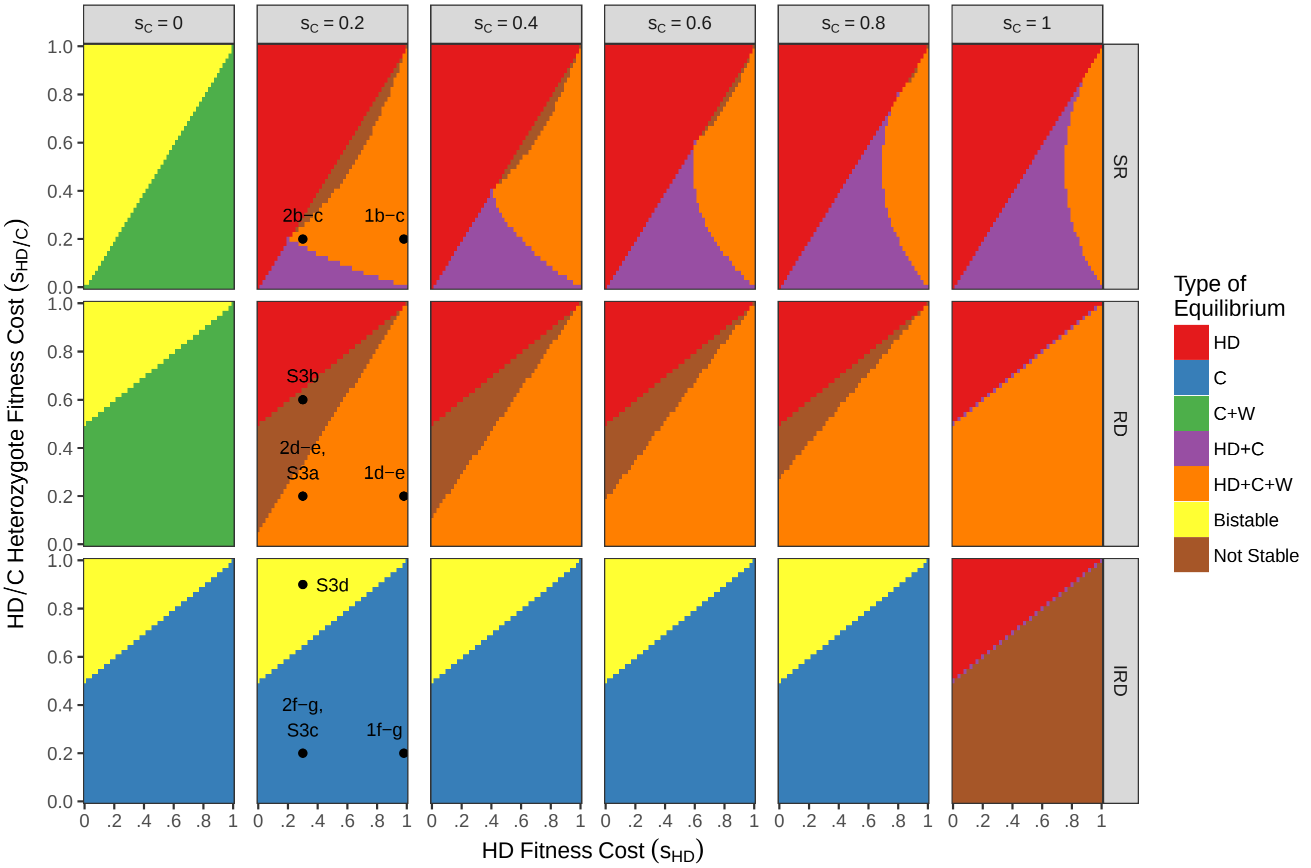

Figure S2. Possible long-term behaviors of the system for each countermeasure and various fitness costs, assuming perfect homing ($e_{HD} = e_C = 1$) and no fitness costs in wild-type heterozygotes ($h_{HD} = h_C = 0$). Colors indicate which alleles are present in the single stable equilibrium of the system (e.g., green indicating that only countermeasure and wild-type are present at equilibrium), if there are multiple stable solutions (yellow), or if there are no stable solutions where all frequencies exist in range [0,1] (brown). The axes show fitness costs of the HD (x-axis) and HD/C heterozygote (y-axis), and the fitness cost of the countermeasure varies across columns. Type of countermeasure varies across rows. Black points indicate parameter combinations used in other figures.

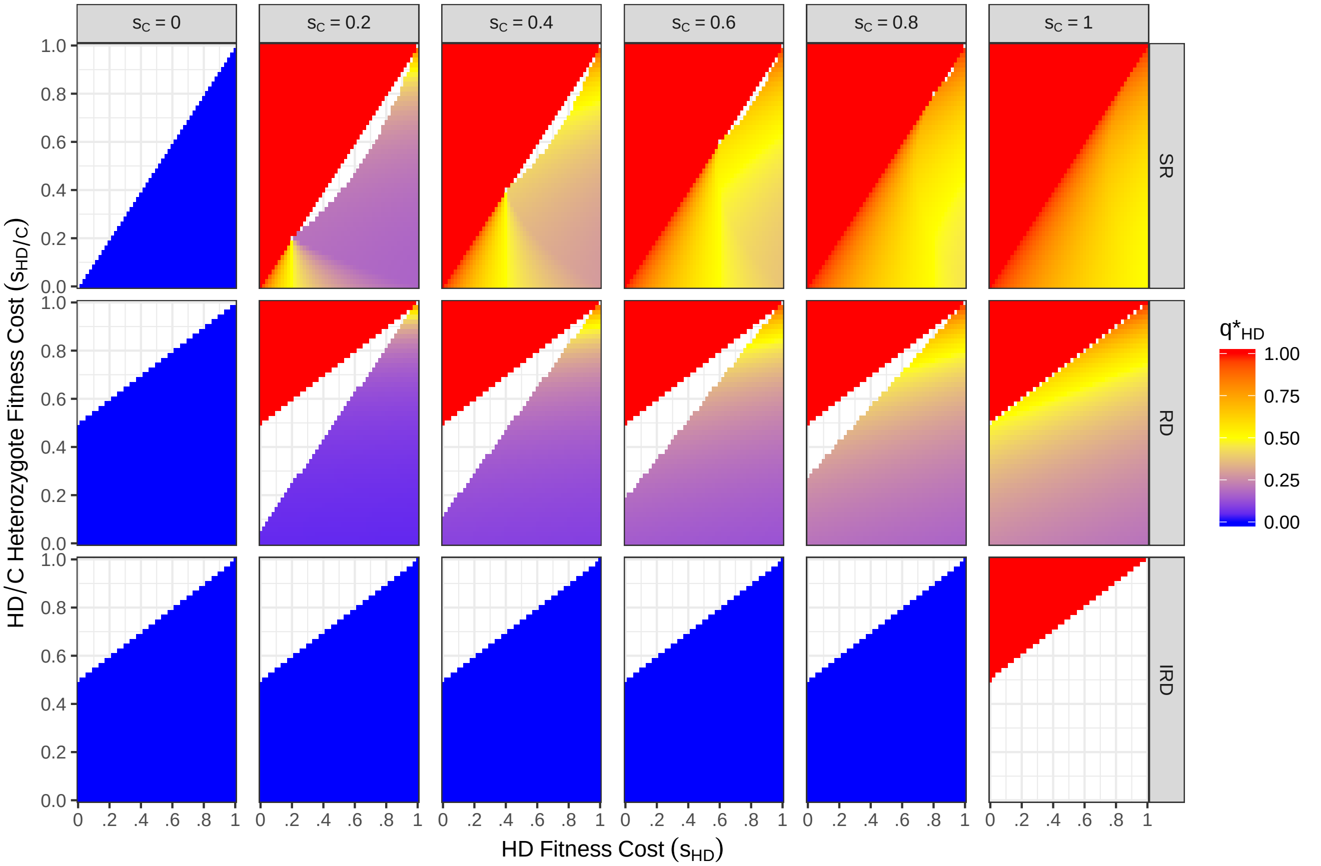

Figure S3. Frequency of the HD at equilibrium for each countermeasure and varying fitness costs, assuming perfect homing ($e_{HD} = e_C = 1$) and recessive fitness costs in wild-type heterozygotes ($h_{HD} = h_C = 0$). Blue and red areas indicate regions of countermeasure and HD fixation, respectively, from Figure S2, and intermediate shades show the HD frequency for stable polymorphic equilibria (orange in Fig. S1). Plot details are otherwise identical to Figure S2.

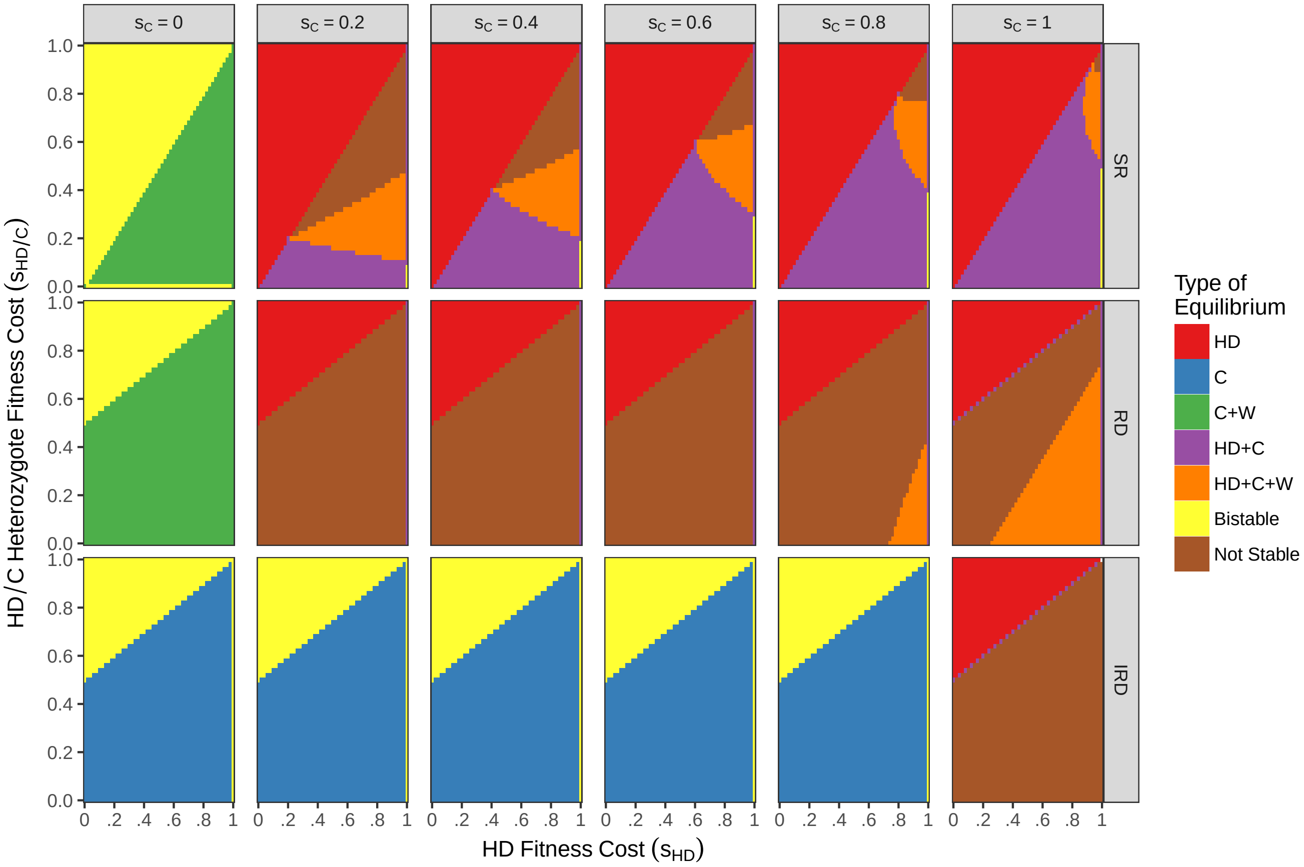

Figure S4. Possible long-term behaviors of the system for each countermeasure and various fitness costs, assuming perfect homing ($e_{HD} = e_C = 1$) and additive fitness costs in wild-type heterozygotes ($h_{HD} = h_C = 0.5$). The axes show fitness costs of the HD (x-axis) and HD/C heterozygote (y-axis), and the fitness cost of the countermeasure varies across columns, and colors indicate the same types of behavior as in Figure S2. Compared with recessive fitness costs in wild-type heterozygotes (Fig. S2), the SR countermeasure becomes more likely to result in removal of wild-type, and the RD countermeasure becomes more likely to have no stable equilibria.

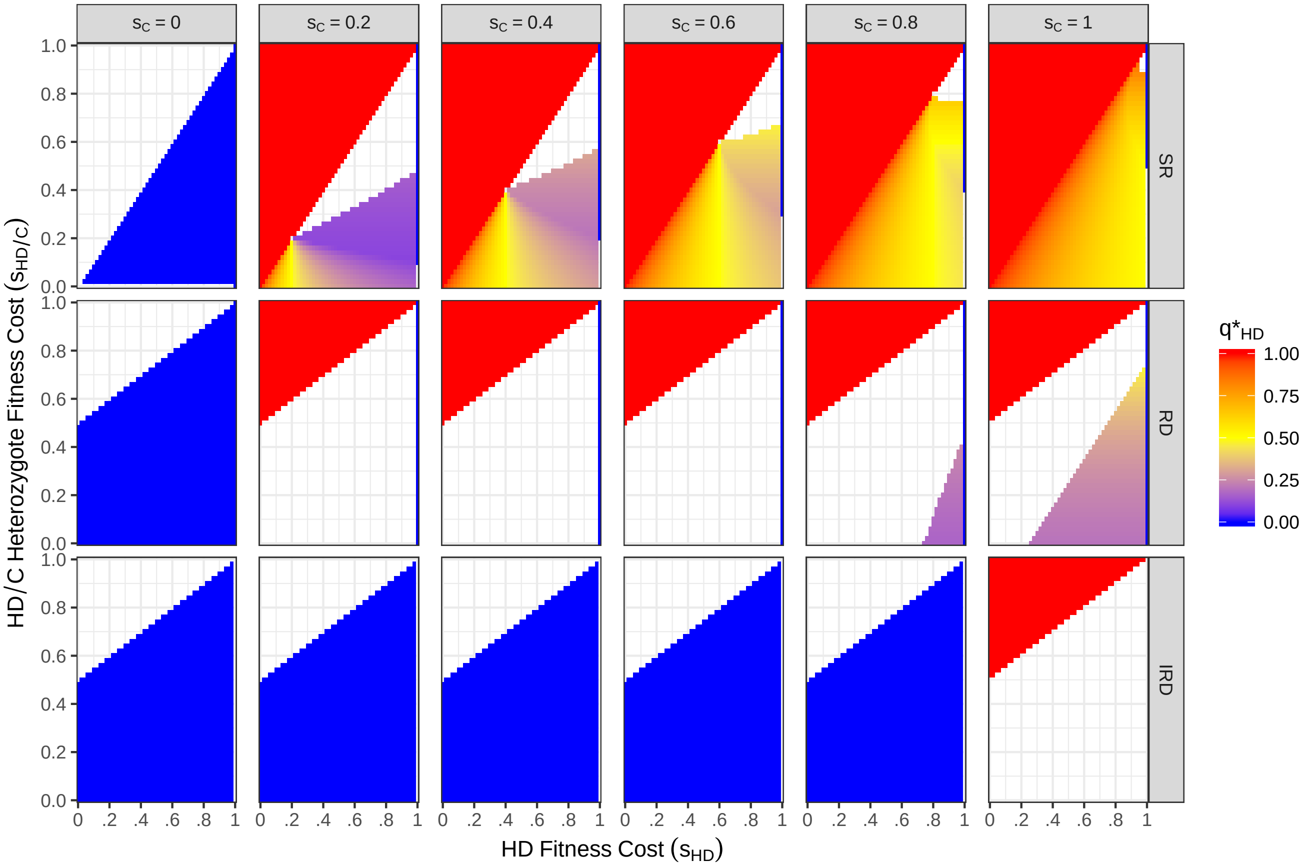

Figure S5. Frequency of the HD at equilibrium for each countermeasure and varying fitness costs, assuming perfect homing ($e_{HD} = e_C = 1$) and additive fitness costs in wild-type heterozygotes ($h_{HD} = h_C = 0.5$). Blue and red areas indicate regions of countermeasure and HD fixation, respectively, from Figure S4, and intermediate shades show the HD frequency for stable polymorphic equilibria (orange in Fig. S4). Plot details are otherwise identical to Figure S4.
